# Supplementary material for: Target-enrichment sequencing yields valuable genomic data for challenging-to-culture bacteria of public health importance
Source: Microb Genom. 2022 May 27;8(5):mgen000836. doi: 10.1099/mgen.0.000836 (PMC9465068; doi:10.1099/mgen.0.000836)
Supplement: Supplementary material 1 [file mgen-8-836-s001.pdf]

Dennis *et al*. Target-enrichment sequencing yields valuable genomic data for challenging-to-culture bacteria of public health importance

Supplementary Material

File S1

Supplementary Methods

Bait Design

For *B. anthracis*, approximately 6 million baits were initially designed; however, after removal of 100% duplicate bait sequences, 2.2 million baits remained, which were queried against the Ames ancestor reference genome using BLASTn to verify hits and remove those that might have been designed across artificial 'joints' in the core genome. This resulted in the removal of 12,796 baits with the longest BLAST hit < 65 bp. To minimize over-tiling towards the 3' end of the alignment (due to staggered bait start positions), baits with 100% identity across more than 75% overlap were clustered, with only one bait per cluster retained (n = 161,899 baits). BLAST queries against targeted mammalian genomes (human, cow, sheep, goat, pig, mouse, rat, camel and donkey) led to the removal of only 3 baits with hits. Baits with >50% simple repeat masking (n=314) were also removed from the bait set. Baits overlapping with any tRNA or rRNA annotations in the reference sequence (n = 171) were removed, as these would be expected to be less specific for *B. anthracis* (i.e. conserved across various bacterial species). Remaining baits were queried against a local database of NCBI RefSeq bacterial genomes using BLASTn, and results parsed with MEGAN6 [1] (min bit score 70, top % 100, min support 0%); baits were removed for any taxonomic rank higher than class Bacilli (i.e. less specific), thus removing a further 1266 baits. Finally, baits with > 98% identity across > 75% overlap were clustered, and 1 bait retained per cluster, effectively removing baits with 1 SNP difference. For *M. amphoriforme*, a total of 25,961 raw baits were designed. Each bait candidate was aligned *in silico* against relevant commensal and pathogenic bacterial genomes (potentially residing in the human respiratory tract and oropharynx; Table S2) and the human genome using BLAST. Baits with hits to these bacterial and human genomes were removed to avoid potential cross-hybridization with exogenous DNA from non-target organisms. Baits were also filtered that were > 25% soft masked for simple repeats.

Sample selection and processing

For *B. anthracis*, samples were stored for up to several months at ambient temperature prior to DNA extraction. DNA was extracted from various sample types using the DNeasy Blood & Tissue kit (Qiagen, UK), with minor modifications to initial sample preparation depending on the sample type as previously described [2]. Final extracts were filtered using a 0.2 µm spin column (Corning Life Sciences) to remove any potentially viable spores prior to downstream molecular analyses. Extracts in this study were from tissues (n = 52), blood swabs (n = 30), whole blood (n

= 9) or insects collected on the carcass (n = 2). Samples were primarily from livestock species, namely sheep (n = 61), cattle (n = 9), goats (n = 5) and a donkey (n = 1), although some samples from wildlife were also included, namely wildebeest (n = 8) and zebras (n = 7); species was not recorded for two samples. Extracted DNA was stored at -20°C (short term) or at -80°C for up to 2 years before being shipped frozen to the UK. All nasopharyngeal swab (NPS) samples collected for *M. amphoriforme* testing were stored at -80 °C. DNA was extracted using Wizard DNA extraction kit (Promega) according to manufacturer's instructions. DNA extracts were stored at -80°C for less than two months, dried using a SpeedVac and shipped at room temperature to the UK. The DNA was reconstituted in nuclease free water and stored at -20°C. Sample-specific metadata for both bacterial species are available as Supplementary Tables A & B at University of Glasgow's Enlighten data repository [3].

Library preparation and bait capture

Samples were prepared in batches of up to 28 samples. Variations made to any experimental parameters are detailed in the supplementary tables on Enlighten [3]. For *B. anthracis*, libraries were prepared following either the Chapter 1 protocol (for inputs ≤ 100 ng) or the Chapter 2 protocol (for inputs ≥ 100 ng) of the NEBNext® Ultra II FS DNA Library Prep Kit; Chapter 1 was followed for all *M. amphoriforme* samples. If initial concentrations of DNA extracts were higher than recommended for the library preparation protocols (i.e. > 100 ng if using Chapter 1, or > 500 ng if using Chapter 2), DNA was diluted in 0.1X TE buffer to achieve the recommended maximum. Dual index NEBNext Multiplex Oligos were used throughout, with the exception that single index primers were used during a trial run with one *B. anthracis* sample. Enzymatic fragmentation was done by incubating at 37°C for 8 minutes, except for *B. anthracis* library batch 5, where incubation was for 10 minutes. For *B. anthracis* NEB library batch 1, 5 amplification cycles were used. Since this resulted in a high proportion of pre-capture libraries with concentrations below the recommended input for bait capture, i.e. 100 ng in 7 µl, from Batch 2 onwards 6 amplification cycles were used, except for Batch 5, where this was increased to 7 cycles. For *M. amphoriforme,* all libraries were prepared using 6 amplification cycles. For *B. anthracis* library batch 1, pre-capture library DNA was eluted in 30 µl of 0.1X TE buffer; from batch 2 onwards, elution was in 23 µl to increase the concentration. For some pre-capture libraries with low initial concentrations as measured on Qubit, libraries were further concentrated prior to bait capture using the Zymo DNA Clean & Concentrator -5 kit (Cambridge Bioscience, UK) enabling elution in very small volumes (i.e. 10 µl). Some captured libraries in *B. anthracis* batches 1 and 2 (in which only 12 amplification cycles were implemented post-hybridisation) had very low concentration, and were therefore subjected to a further 5 PCR amplification cycles, followed by a second clean-up step.

Bioinformatics

After trimmed reads were mapped using bwa-mem, duplicate reads were removed, and read groups added with *gatk* v4.2.1.0 MarkDuplicates and AddOrReplaceReadGroups as part of their standard pipeline [4]. Mapping and coverage statistics were collected by calculating coverage over baited regions using bait .bed annotation files in *gatk DepthOfCoverage*. Mean depth-of-coverage was calculated as: number mapped reads * read length / length of the baited reference genome. Samtools v1.3.1 *flagstat* was used to collect data on the number of duplicate reads [5]. The workflow was implemented in *nextflow* v20.10.0.5430 [6]. Scripts for the analysis of both datasets were written in R [7], incorporating *tidyverse* v1.3.0 [8], *DHARMa* v0.4.3 [9] and *cowplot* v1.1.1 [10]. The *nextflow* workflow, Docker container link, and analysis scripts are available at https://github.com/tristanpwdennis/bactocap. For each organism, for 10 arbitrarily selected samples, Kraken 2 [11] was run against the MiniKraken 8GB database to identify the taxonomic designation of sample reads.

Supplementary Results

Ct values of sequenced samples

Taking the highest of the *Ct* values per sample from qPCR of the three *B. anthracis* genomic targets, the median was 25 across the 93 samples; this was the same for both the group of pooled and unpooled samples. The variance in *Ct* for unpooled samples was 21.9, and for pooled samples was 4.9. The median (and variance) of *Ct* values of qPCR from *M. amphoriforme* was 29 (8.3), whereas it was 36 (5.6) for pooled samples. Sample-specific sequencing results are available on Enlighten [3].

Captured library concentrations

Following bait capture, concentrations of captured *B. anthracis* libraries ranged from 0.16 to 66 ng/µl, with a median post-capture library concentration of 1.6 ng/µl (variance = 166.3). For *M. amphoriforme,* captured library concentrations ranged from 0.2 to 3.3 ng/µl, with a median of 0.8 ng/µl (variance = 0.58).

Provenance of unmapped reads

The results from Kraken 2 suggest that most unmapped reads likely corresponded to host material. For the *M. amphoriforme* dataset, the largest proportion of reads were assigned to the genus *Homo* (i.e. human origin) (Table S5); unclassified reads are expected to be primarily *M. amphoriforme,* which is not part of the MiniKraken database. For the *B. anthracis* dataset, reads were mostly assigned to *Bacillus* (Table S6); unclassified reads are likely to be different ruminant hosts, again not part of the MiniKraken database.

Supplementary Figures

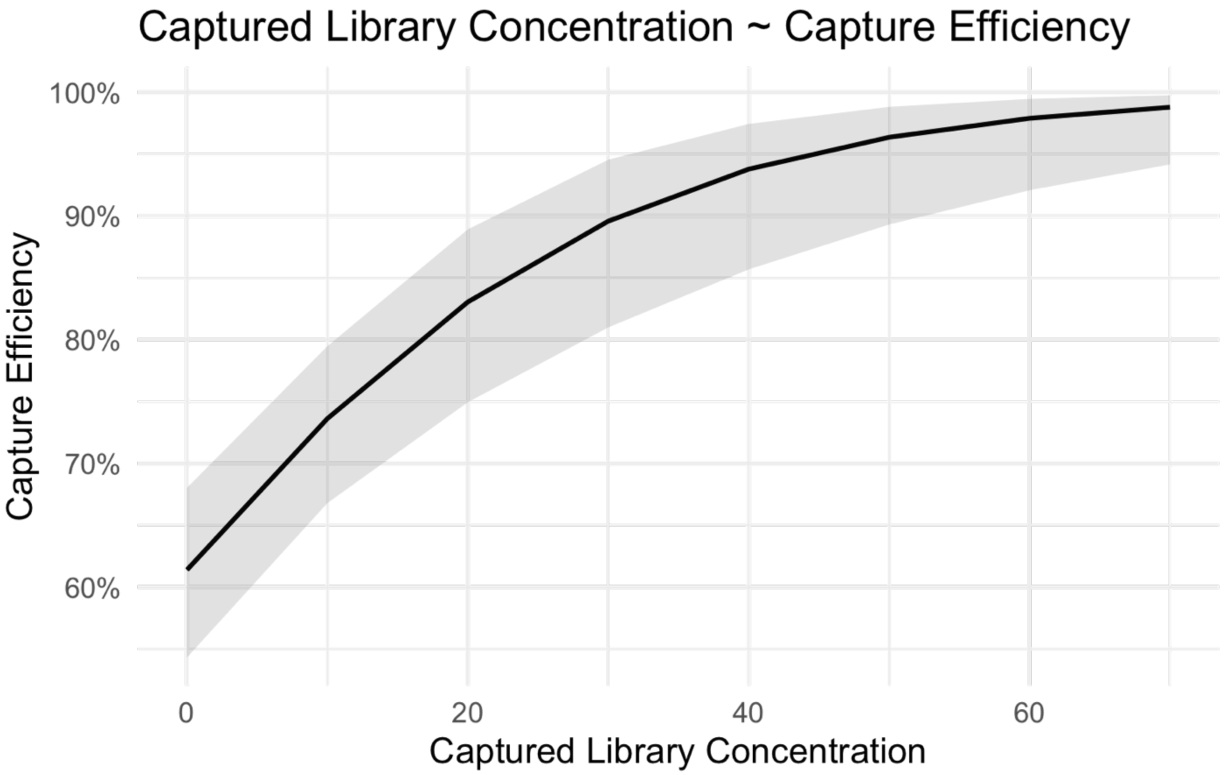

Fig. S1. Predicted relationship between captured library concentration and capture efficiency for both species. Shading represents a 95% prediction interval.

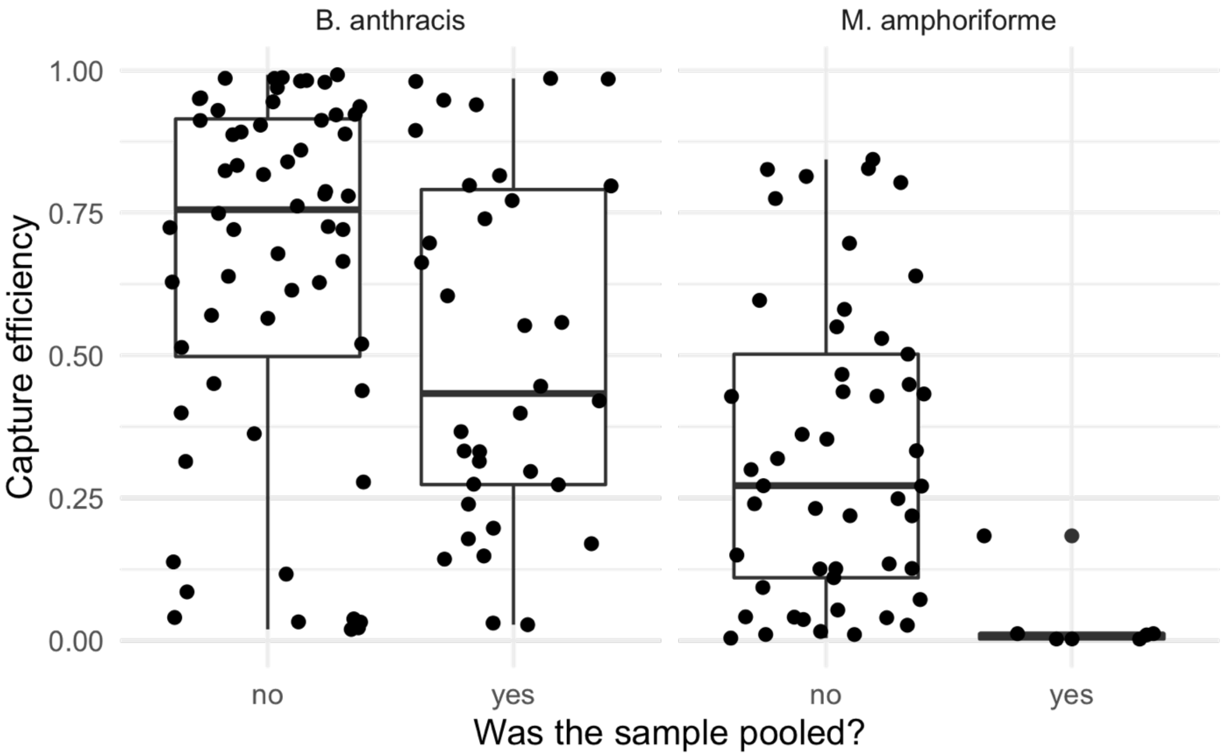

Fig. S2. Box-and-whisker plots overlaid with jittered points indicating capture efficiency vs whether the sample was pooled or unpooled prior to bait capture. Shown for both *B. anthracis* and *M. amphoriforme.*

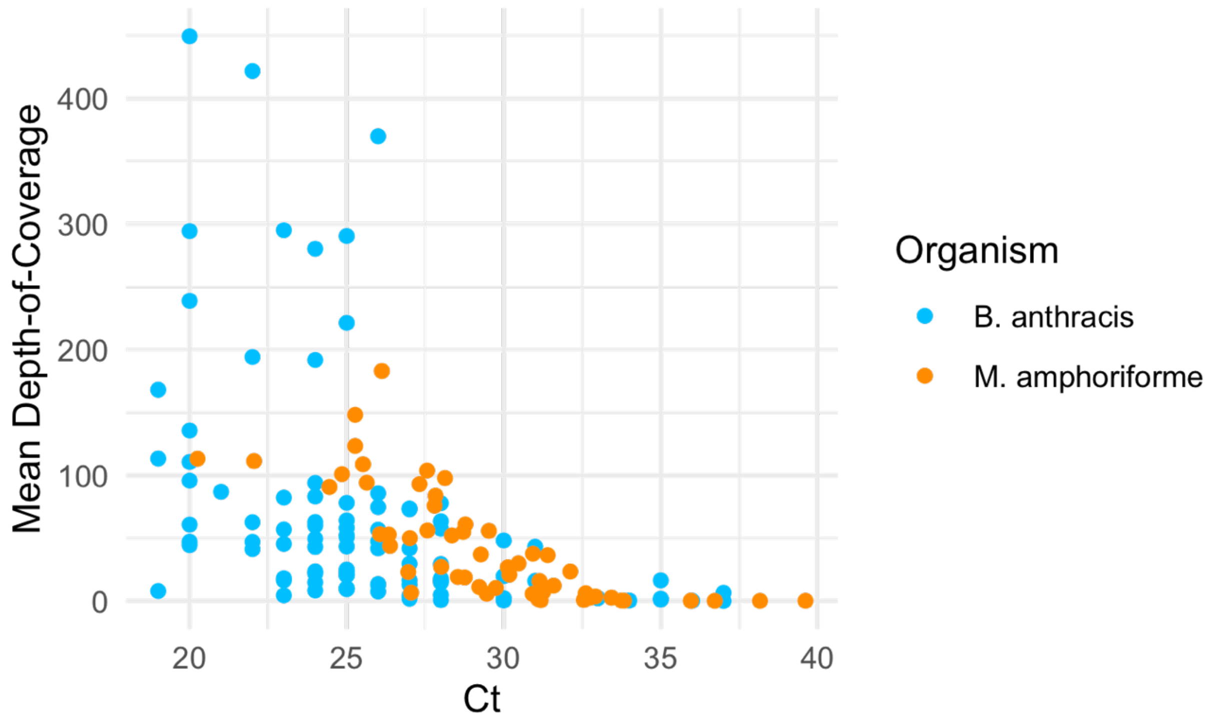

Fig. S3. Relationship between *Ct* value and mean depth-of-coverage. Each point represents an individual sample, with bacterial species distinguished by colour.

Supplementary Tables

Table S1. Publicly available *Bacillus anthracis* genome sequences used to generate a core genome alignment for the design of *B. anthracis* specific baits.

Strain	BioSample	BioProject	Assembly	NCBI Reference Sequence
2000031021	SAMN02736984	PRJNA243523	GCA_000742655.1	NZ_CP007618.1/CP007618.1
2002013094	SAMN03174509	PRJNA238050	GCA_000832965.1	NZ_CP009902.1/CP009902.1
HYU01	SAMN02874036	PRJNA231762	GCA_000725325.1	NZ_CP008846.1/CP008846.1
SVA11	SAMN03081486	PRJNA217316	GCA_000583105.1	NZ_CP006742.1/CP006742.1
BA1035	SAMN03010427	PRJNA238135	GCA_000832725.1	NZ_CP009700.1/CP009700.1
RA3	SAMN03075602	PRJNA238136	GCA_000832745.1	NZ_CP009697.1/CP009697.1
Tyrol 4675	SAMN06186720	PRJNA309927	GCA_001936375.1	NZ_CP018903.1/CP018903.1
K3	SAMN03010428	PRJNA238080	GCA_000832465.1	NZ_CP009331.1/CP009331.1
H9401	SAMN02603474	PRJNA49361	GCA_000258885.1	NC_017729.1/CP002091.1
SK-102	SAMN03012770	PRJNA238068	GCA_000832565.1	NZ_CP009464.1/CP009464.1

CDC 684	SAMN02603931	PRJNA31329	GCA_000021445.1	NC_012581.1/CP001215.1
Vollum	SAMN02736982	PRJNA243521	GCA_000742895.1	NZ_CP007666.1/CP007666.1
Vollum 1B	SAMN03010433	PRJNA238082	GCA_000832445.1	NZ_CP009328.1/CP009328.1
Pasteur	SAMN03024436	PRJNA238046	GCA_000832585.1	NZ_CP009476.1/CP009476.1
Smith 1013	SAMN02732407	PRJNA243516	GCA_000742315.1	NZ_CM002879.1/CM002879.1
A0157	SAMN03267488	PRJNA270580	GCA_000808075.1	NZ_CP010342.1/CP010342.1
Turkey32	SAMN03010432	PRJNA236040	GCA_000833275.1	NZ_CP009315.1/CP009315.1
PAK-1	SAMN03010430	PRJNA237808	GCA_000832425.1	NZ_CP009325.1/CP009325.1
A1144	SAMN02999504	PRJNA257008	GCA_000875715.1	NZ_CP010852.1/CP010852.1
Canadian_bison	SAMN03202901	PRJNA238044	GCA_000833125.1	NZ_CP010322.1/CP010322.1
Pollino	SAMN03296000	PRJNA273788	GCA_000831505.1	NZ_CP010813.1/CP010813.1
Larissa	SAMN03765650	PRJNA286154	GCA_001277955.1	NZ_CP012519.1/CP012519.1
BA1015	SAMN03010426	PRJNA238204	GCA_000832665.1	NZ_CP009544.1/CP009544.1
V770-NP-1R	SAMN03092715	PRJNA235226	GCA_000832785.1	NZ_CP009598.1/CP009598.1
Ohio ACB	SAMN03010429	PRJNA238205	GCA_000832505.1	NZ_CP009341.1/CP009341.1
52-G	SAMN02951870	PRJNA224563	GCA_000559005.1	NZ_CM002395.1/CM002395.1
8903-G	SAMN02951868	PRJNA224562	GCA_000558965.1	NZ_CM002401.1/CM002401.1
9080-G	SAMN02951869	PRJNA224558	GCA_000558985.1	NZ_CM002398.1/CM002398.1
A16	SAMN02641483	PRJNA40303	GCA_000512835.2	NZ_CP001970.2/CP001970.2
Ames	SAMN02603432	PRJNA309	GCA_000007845.1	NC_003997.3/AE016879.1
Ames Ancestor	SAMN02603433	PRJNA10784	GCA_000008445.1	NC_007530.2/AE017334.2
A0248	SAMN02603932	PRJNA33543	GCA_000022865.1	NC_012659.1/CP001598.1
Ames	SAMN03201418	PRJNA238045	GCA_000833065.1	NZ_CP009981.1/CP009981.1
Stendal	SAMN04442145	PRJNA309927	GCA_001543225.1	NZ_CP014179.1/CP014179.1
14RA5914	SAMN07498358	PRJNA397960	GCA_002277915.1	NZ_CP023001.1/CP023001.1
Tangail-1	SAMN05003865	PRJNA309927	GCA_001654475.1	NZ_CP015779.1/CP015779.1
BFV	SAMN02736972	PRJNA243518	GCA_000742875.1	NZ_CP007704.1/CP007704.1
Sterne	SAMN03010431	PRJNA236483	GCA_000832635.1	NZ_CP009541.1/CP009541.1
Sterne	SAMN02598266	PRJNA10878	GCA_000008165.1	NC_005945.1/AE017225.1
delta Sterne	SAMN02736981	PRJNA243519	GCA_000742695.1	NZ_CP008752.1/CP008752.1

Parent1	SAMN04093743	PRJNA295544	GCA_001683095.1	NZ_CP012730.1/CP012730.1
SPV842_15	SAMN06270326	PRJNA368680	GCA_001990245.1	NZ_CP019588.1/CP019588.1
PR02	SAMN04075677	PRJNA295544	GCA_001683155.1	NZ_CP012721.1/CP012721.1
PR06	SAMN04075681	PRJNA295544	GCA_001683195.1	NZ_CP012723.1/CP012723.1
PR01	SAMN04075676	PRJNA295544	GCA_001683135.1	NZ_CP012720.1/CP012720.1
PR05	SAMN04075680	PRJNA295544	GCA_001683175.1	NZ_CP012722.1/CP012722.1
PR07	SAMN04075682	PRJNA295544	GCA_001683215.1	NZ_CP012724.1/CP012724.1
PR09-1	SAMN04075684	PRJNA295544	GCA_001683255.1	NZ_CP012726.1/CP012726.1
Parent2	SAMN04093744	PRJNA295544	GCA_001683065.1	NZ_CP012729.1/CP012729.1
PR08	SAMN04075683	PRJNA295544	GCA_001683235.1	NZ_CP012725.1/CP012725.1
PR09-4	SAMN04075685	PRJNA295544	GCA_001683275.1	NZ_CP012727.1/CP012727.1
PR10-4	SAMN04075686	PRJNA295544	GCA_001683295.1	NZ_CP012728.1/CP012728.1

Table S2. Human respiratory commensal and pathogenic bacterial genome sequences used for the design of *M. amphoriforme* specific baits. Non-specific baits with BLAST hits to these organisms were removed.

Accession number	Strain
NC_002929	*Bordetella pertussis Tohama I*
NZ_CP012981	*Burkholderia cepacia ATCC 25416, chromosome 1*
NZ_CP012982	*Burkholderia cepacia ATCC 25416, chromosome 2*
NZ_CP012983	*Burkholderia cepacia ATCC 25416, chromosome 3*
NZ_LN831026	*Corynebacterium diphtheriae NCTC11397*
NZ_ACEA00000000	*Eikenella corrodens ATCC 23834*
NZ_AJSY00000000	*Fusobacterium necrophorum*
NC_000907	*Haemophilus influenzae Rd KW20*
NC_015964	*Haemophilus parainfluenzae T3T1*
NC_014147	*Moraxella catarrhalis BBH18*
NC_000962	*Mycobacterium tuberculosis H37Rv*
NC_000908	*Mycoplasma genitalium G37*
NC_013511	*Mycoplasma hominis ATCC 23114*
NZ_ATUH00000000	*Mycoplasma orale ATCC 23714*
NC_000912	*Mycoplasma pneumoniae M129*
NZ_AXZE00000000	*Mycoplasma salivarium ATCC 23064*
NZ_CP007726	*Neisseria elongata subsp. glycolytica ATCC 29315*
NC_014752	*Neisseria lactamica 020-06*
NC_003112	*Neisseria meningitidis MC58*
NC_010729	*Porphyromonas gingivalis ATCC 33277*
NC_014370	*Prevotella melaninogenica ATCC 25845, chromosome 1*
NC_014371	*Prevotella melaninogenica ATCC 25845, chromosome 2*
NZ_AEPE00000000	*Prevotella oralis ATCC 33269*
NZ_ARIR00000000	*Prevotella veroralis DSM 19559 = JCM 6290*

NC_002516	*Pseudomonas aeruginosa PAO1*
NZ_HG326223	*Serratia marcescens subsp. marcescens Db11*
NC_007795	*Staphylococcus aureus subsp. aureus NCTC 8325*
NC_004461	*Staphylococcus epidermidis ATCC 12228*
NC_013853	*Streptococcus mitis B6*
NC_004350	*Streptococcus mutans UA159*
NC_015291	*Streptococcus oralis Uo5*
NC_003098	*Streptococcus pneumoniae R6*
NC_015875	*Streptococcus pseudopneumoniae IS7493*
NC_002737	*Streptococcus pyogenes M1 GAS*
NZ_CP009913	*Streptococcus salivarius NCTC 8618*
NC_009009	*Streptococcus sanguinis SK36*
NC_010503	*Ureaplasma parvum serovar 3 str. ATCC 27815*
NC_011374	*Ureaplasma urealyticum serovar 10 str. ATCC 33699*
NC_013520	*Veillonella parvula DSM 2008*

Table S3. Summary statistics. Total reads, mapped reads, fraction of mapped reads, mean depth of coverage (doc), fraction of the genome covered > 15X, for *B. anthracis* and *M. amphoriforme.*

	B. anthracis	*M. amphoriforme*
median_total_reads	6413796	2989985
max_total_reads	32708157	4247924
min_total_reads	539623	1082454
mean_total_reads	7624145	2918092
median_mapped_reads	3589129	738985
max_mapped_reads	32291415	3056077
min_mapped_reads	29490	10390
mean_mapped_reads	5442403	836116
median_frac_mapped_reads	0.697	0.236
max_frac_mapped_reads	0.992	0.844
min_frac_mapped_reads	0.023	0.003
mean_frac_mapped_reads	0.611	0.294
median_meandoc	43.19	26.95
max_meandoc	449.39	183.05
min_meandoc	0.17	0.08
mean_meandoc	69.66	42.91
iqr_meandoc	59.96	58.93
upper_quartile_meandoc	74.73	64.68
lower_quartile_meandoc	14.77	5.76
median_frac_above15	97.3	79.2
max_frac_above15	100	95.8
min_frac_above15	0	0
mean_frac_above15	69.27	55.61
iqr_frac_above15	55.9	88.6
upper_quartile_frac_above15	99.8	90.8
lower_quartile_frac_above15	43.9	2.15

Table S4. Model details for binomial glmm of the effect of *Ct* value, captured library concentration and pooling prior to bait capture on capture efficiency, including an individual-level random effect.

**Results of binomial *glmm***	**Estimate**	**Std. Error**	**z value**	**Pr(>|z|)**
(Intercept)	9.66832	0.78245	12.357	< 2e-16 ***
max_ct	-0.35679	0.02788	-12.799	< 2e-16 ***
cap_lib_conc	0.05805	0.01231	4.715	2.42e-06 ***
pooled_yesorno	-1.31290	0.27892	-4.707	2.42e-06 ***

***drop1* output:**	**npar**	**AIC**	**LRT**	**Pr(Chi)**
<none>		4451.8		
max_ct	1	4541.5	110.716	< 2.2e-16 ***
cap_lib_conc	1	4451.5	20.726	5.299e-06 ***
pooled_yesorno	1	4451.4	20.666	5.468e-06 ***

***MuMIn* output:**		**R2m**	**R2c**
	theoretical	0.6124126	0.9999999
	delta	0.6124126	0.9999999

Table S5: Kraken 2 output for 10 arbitrarily selected *M. amphoriforme samples*. Only genera corresponding to >1% of the total read count are displayed.

Sample	Classification (Genus)	% Total Reads
07B00774_S16	unclassified	4.67
07B00774_S16	Homo	94.61
08B00436_S7	unclassified	8.72
08B00436_S7	Homo	90.58
08B02193_S8	unclassified	6.17
08B02193_S8	Homo	93.53
08B03746_S9	unclassified	37.08
08B03746_S9	Homo	55.86
08B03746_S9	Lactococcus	4.9
08B04270_S10	unclassified	15.66
08B04270_S10	Homo	82.09
08B04270_S10	Lactococcus	1.57
08B05877_S72	unclassified	30.39
08B05877_S72	Homo	69.31
08B06178_S73	unclassified	47.11
08B06178_S73	Homo	52.47
08B07005_S51	unclassified	13.91
08B07005_S51	Homo	85.12
08B07142_S58	unclassified	38.01
08B07142_S58	Homo	60.68
08B07429_S46	unclassified	9.92
08B07429_S46	Homo	89.43

Table S6: Kraken 2 output for 10 arbitrarily selected *B. anthracis samples*. Only genera corresponding to >1% of the total read count are displayed.

Sample	Classification (Genus)	% total reads
AN16-06-B_S14	unclassified	5.53
AN16-06-B_S14	Bacillus	93.5
AN16-06-I_S29	unclassified	45.55
AN16-06-I_S29	Bacillus	39.95
AN16-06-I_S29	Lysinibacillus	1.32
AN16-06-I_S29	Vagococcus	1.02
AN16-06-I_S29	Providencia	3.12
AN16-06-I_S29	Psychrobacter	1.97
AN16-06-T_S4	unclassified	15.98
AN16-06-T_S4	Bacillus	77.59
AN16-06-T_S4	Clostridium	1.68
AN16-06-T_S4	Proteus	2.44
AN16-120-S_S23	unclassified	5.5
AN16-120-S_S23	Bacillus	93.82
AN16-130-S_S36	unclassified	72.99
AN16-130-S_S36	Bacillus	5.67
AN16-130-S_S36	Macrococcus	3.82
AN16-130-S_S36	Acinetobacter	5.71
AN16-130-S_S36	Paenalcaligenes	1.49
AN16-130-T_S25	unclassified	8.64
AN16-130-T_S25	Bacillus	90.79
AN16-132-I_S32	unclassified	44.13
AN16-132-I_S32	Bacillus	39.05
AN16-132-I_S32	Lysinibacillus	2.14
AN16-132-I_S32	Staphylococcus	1.06
AN16-132-I_S32	Vagococcus	3.81
AN16-135-2-T_S1	unclassified	13.11
AN16-135-2-T_S1	Bacillus	86.22
AN16-144-S_S40	unclassified	8.12
AN16-144-S_S40	Bacillus	88.9
AN16-144-S_S40	Acinetobacter	1.77
AN16-145-B_S1	unclassified	4.12
AN16-145-B_S1	Bacillus	95.44

Supplementary References

1. **Huson DH, Beier S, Flade I, Górska A, El-Hadidi M, *et al.*** MEGAN Community Edition - Interactive Exploration and Analysis of Large-Scale Microbiome Sequencing Data. *PLOS Computational Biology* 2016;12:e1004957.

2. **Aminu OR, Lembo T, Zadoks RN, Biek R, Lewis S, *et al.*** Practical and effective diagnosis of animal anthrax in endemic low-resource settings. *PLOS Neglected Tropical Diseases* 2020;14:e0008655.

3. **Dennis TPW, Mable B, Brunelle B, Devault A, Carter R, *et al.*** Supplementary Tables A & B. http://dx.doi.org/10.5525/gla.researchdata.1249 (2022).

4. **Van der Auwera GA, O'Connor BD**. *Genomics in the Cloud*. O'Reilly Media, Inc; 2020.

5. **Li H, Handsaker B, Wysoker A, Fennell T, Ruan J, *et al.*** The Sequence Alignment/Map format and SAMtools. *Bioinformatics* 2009;25:2078–2079.

6. **Di Tommaso P, Chatzou M, Floden EW, Barja PP, Palumbo E, *et al.*** Nextflow enables reproducible computational workflows. *Nat Biotechnol* 2017;35:316–319.

7. **R Core Team**. *R: A language and environment for statistical computing*. Vienna, Austria: R Foundation for Statistical Computing. https://www.R-project.org/ (2021).

8. **Wickham H, Averick M, Bryan J, Chang W, McGowan LD, *et al.*** Welcome to the Tidyverse. *Journal of Open Source Software* 2019;4:1686.

9. **Hartig F**. *DHARMa: residual diagnostics for hierarchical (multi-level/mixed) regression models*. https://cran.r-project.org/web/packages/DHARMa/vignettes/DHARMa.html.

10. **Wilke CO**. *cowplot – Streamlined plot theme and plot annotations for ggplot2*. https://wilkelab.org/cowplot/.

11. **Wood DE, Lu J, Langmead B**. Improved metagenomic analysis with Kraken 2. *Genome Biol* 2019;20:1–13.
